# Occurrence, Risk Factors, and Molecular Characterization of *Ehrlichia canis* Infection in Clinically Suspected Dogs from a Tropical Region of South India

**DOI:** 10.3390/vetsci13060568

**Published:** 2026-06-09

**Authors:** Jalajakshi Kopparthi, Sreedevi Chennuru, Chengalva Rayulu Vukka, Karumuri Nalini Kumari, Devalam Rani Prameela, Ravikanthreddy Poonooru

**Affiliations:** 1 College of Veterinary Science, Sri Venkateswara Veterinary University, Tirupati 517 502, India; jalajakshikopparthi@gmail.com (J.K.);; 2 NTR College of Veterinary Science, Sri Venkateswara Veterinary University, Gannavaram 521 102, India; 3 College of Veterinary Science, Sri Venkateswara Veterinary University, Garividi 535 101, India; 4 Division of Animal Sciences, University of Missouri, Columbia, MO 65211, USA

**Keywords:** tick-borne disease, dogs, *Ehrlichia canis*, PCR, microscopy, epidemiology

## Abstract

Canine monocytic ehrlichiosis, caused by *Ehrlichia canis*, is an important tick-borne disease of dogs in tropical and subtropical regions. This cross-sectional study investigated the occurrence, associated risk factors, and molecular confirmation of *E. canis* infection among clinically suspected dogs in Andhra Pradesh, India, and evaluated the diagnostic performance of blood smear microscopy relative to PCR. Peripheral blood samples were collected from 442 clinically suspected dogs presented to 90 veterinary dispensaries across the state. Dogs were selected based on clinical features suggestive of ehrlichiosis, including inappetence, pale mucous membranes, tick infestation, fever, lymphadenopathy, weakness, and hemorrhagic signs. Samples were examined by Giemsa-stained blood smear microscopy and subsequently tested by PCR. Representative *virB9* gene amplicons were sequenced and compared with reference sequences available in GenBank. Microscopy detected *E. canis* in 37/442 dogs (8.37%), whereas PCR detected infection in 51/442 dogs (11.54%). Logistic regression analysis indicated that female dogs, mongrels, kennel dogs, and dogs with tick infestation had significantly higher odds of infection, while dogs with a history of tick-control measures had lower odds. Sequence comparison and phylogenetic placement based on the partial *virB9* fragment supported molecular confirmation of the detected *E. canis* isolates and showed similarity with previously reported Indian and international isolates. These findings provide regional evidence on the occurrence and epidemiology of canine ehrlichiosis in clinically suspected dogs and support PCR as a more reliable confirmatory method than microscopy.

## Simple Summary

Canine monocytic ehrlichiosis is an important tick-borne disease of dogs caused by *Ehrlichia canis*. It is commonly seen in tropical and subtropical areas where the brown dog tick is widely present. The present study investigated clinically suspected dogs from Andhra Pradesh, India, to better understand the occurrence of this infection, the major risk factors associated with it, and the characteristics of the circulating pathogen. Blood samples from 442 dogs presented to 90 veterinary dispensaries were examined by microscopy and PCR. PCR detected more positive cases than microscopy, showing its greater usefulness as a confirmatory test under field conditions. The study also found that female dogs, mongrels, kennel dogs, and dogs with tick infestation had higher odds of infection, whereas dogs with a history of tick-control measures showed lower odds. In addition, sequence analysis of the *virB9* gene confirmed the identity of the detected isolates and showed genetic similarity with previously reported Indian and international isolates. These findings provide useful regional information on canine ehrlichiosis and may support improved diagnosis, surveillance, and tick-control strategies in similar tropical settings.

## 1. Introduction

Canine monocytic ehrlichiosis is an important tick-borne disease of dogs caused by the obligate intracellular bacterium *Ehrlichia canis* [1]. In India, the disease is clinically important because tropical and subtropical climatic conditions favor the persistence of the brown dog tick, *Rhipicephalus sanguineus* sensu lato, the principal vector of *E. canis*. Dogs in many regions are frequently exposed to ticks, and diagnosis under routine veterinary conditions can be challenging because clinical signs are often nonspecific and access to molecular testing may be limited. Although *E. canis* is primarily recognized as a canine pathogen, occasional reports of human infection have raised concern regarding its potential zoonotic relevance [2]. Because the survival, reproduction, and activity of *R. sanguineus* are influenced by environmental factors such as temperature and humidity, the occurrence of canine ehrlichiosis may vary across geographic locations [3]. Therefore, region-specific information on disease occurrence and associated risk factors is important for surveillance, diagnosis, and tick-control planning.

In clinical practice, diagnosis of canine ehrlichiosis often relies on a combination of history, clinical findings, and laboratory testing. Microscopic examination of Giemsa-stained blood smears remains one of the simplest and most accessible methods for routine veterinary use, particularly in resource-limited and field-oriented settings. However, its diagnostic utility is limited in subclinical, chronic, or carrier animals because circulating morulae may be few, transient, or difficult to detect. In addition, microscopy cannot provide molecular confirmation of the infecting organism. Although PCR is widely recognized as a more sensitive confirmatory method, microscopy continues to be used in many rural and semi-urban veterinary facilities where molecular testing is not routinely available. Therefore, evaluating the practical diagnostic yield of microscopy in comparison with PCR-based confirmation remains relevant for routine veterinary practice.

Despite the clinical importance of canine ehrlichiosis, region-specific data on its occurrence, associated risk factors, and molecular confirmation in clinically suspected dog populations remain limited in several parts of India, including Andhra Pradesh. Field-based studies that integrate diagnostic comparison, risk-factor analysis, and sequence-based characterization are useful for understanding local epidemiology and improving practical control strategies. Accordingly, the present study was designed to address three related objectives: first, to determine the occurrence of *E. canis* among clinically suspected dogs in Andhra Pradesh; second, to evaluate major host- and management-related risk factors associated with infection; and third, to compare the diagnostic yield of microscopy with PCR and confirm and compare representative *E. canis* amplicon sequences using the partial *virB9* gene.

## 2. Materials and Methods

### 2.1. Ethical Approval

The current study was approved by the Institute’s animal ethics committee (IAEC), College of Veterinary Science, Sri Venkateswara Veterinary University, as per the rules and guidelines framed and communicated by the Committee for the Purpose of Control and Supervision of Experiments on Animals (CPCSEA), a statutory committee established under Chapter 4, Section 15(1) of the Prevention of Cruelty to Animals Act 1960, India (CPCSEA/IAEC/SVVU/VPA-2018).

### 2.2. Study Location and Dogs

**Figure 1:**
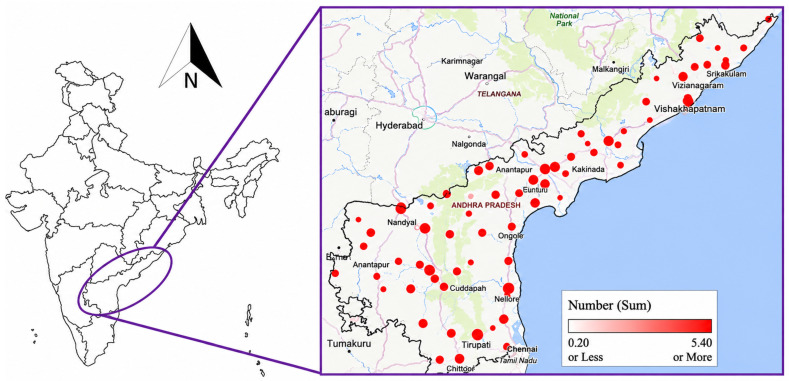
Map of the study area and locations of sample collection. The map on the left side shows the Indian sub-continent and the map on right shows Andhra Pradesh (AP) province. A red dot indicates the location of a sample site (veterinary dispensary or clinical complex) within the AP province and the variation in transparency indicates the total number of samples collected within the site.

Dogs were included in the study when they were clinically suspected of canine ehrlichiosis based on one or more compatible clinical findings, including inappetence, pale conjunctival mucous membranes, visible tick infestation, fever, enlarged popliteal lymph nodes, prolonged weakness, petechiae, ecchymoses, epistaxis, lameness, dyspnea, limb edema, scrotal edema, melena, hematuria, ataxia, twitching, hyperesthesia, or paresis. The dogs were selected from 90 veterinary dispensaries covering all 13 districts of Andhra Pradesh, South India (12°41′ to 19.07° N latitude and 77° to 84°40′ E longitude) (Figure 1). The climate of the region is generally hot and humid, with a mean annual temperature of 33.2 °C and humidity ranging from 66% to 84% [4,5].

### 2.3. Sample and Data Collection

#### 2.3.1. Blood Samples and Tick Collection

Peripheral blood samples were collected from clinically suspected dogs using two separate approaches. Capillary blood was obtained by ear puncture to allow the immediate preparation of fresh thin blood smears for microscopic examination, thereby minimizing artifacts associated with delayed smear preparation or anticoagulant exposure. Venous blood was collected aseptically from the cephalic vein into EDTA-coated vials and stored at −20 °C until DNA extraction and PCR analysis. Each dog was also thoroughly examined for ticks by inspecting the skin and hair coat. When present, ticks were collected and preserved in 70% ethanol for later identification using the taxonomic keys of Walker et al. [6].

#### 2.3.2. Data Collection for Risk Factor Analysis

A range of canine demographic and environmental factors were investigated to identify potential risk factors associated with the occurrence of Ehrlichiosis in Andhra Pradesh, South India. Each factor was chosen based on its relevance to the canine population and its potential impact on the susceptibility to Ehrlichiosis. The diverse risk factors and the underlying reasoning for their selection encompass:

1. Age (≥one year or ≤one year): Age is a fundamental factor, as younger and older canines may exhibit different levels of susceptibility to Ehrlichiosis. Puppies and geriatric dogs might have distinct immune responses, influencing their vulnerability.
2. Gender (male/female): Investigating the gender factor allows for an exploration of potential hormonal or behavioral influences on Ehrlichiosis occurrence.
3. Breed (purebreds/mongrels): Canine breeds exhibit genetic variations that might influence their resistance or susceptibility to specific diseases such as Ehrlichiosis.
4. Residential setting (urban/rural): Environmental differences between urban and rural settings can impact the exposure of dogs to vectors carrying Ehrlichia. Hence, understanding the role of the living environment is crucial in devising targeted control strategies.
5. Living condition (stray dogs/pet dogs/kennel dogs): The living conditions of canines, whether as strays, pets, or in kennels, can affect their exposure to ticks and other vectors. This factor explores the association between living conditions and the occurrence of Ehrlichiosis.
6. Season (winter/summer/rainy): Seasonal variations influence the abundance and activity of ticks, which are primary vectors of Ehrlichia. Investigating seasonality provides insights into the temporal dynamics of disease transmission.
7. Ticks (present/absent): The presence or absence of ticks directly correlates with the risk of Ehrlichiosis transmission. This factor helps establish a clear link between tick infestation and disease occurrence.
8. Tick control measures (not followed/followed): The implementation of tick control measures is a crucial aspect of preventing Ehrlichiosis. Examining whether these measures are followed or not provides insights into the effectiveness of preventive strategies.

A complete database for each dog was developed by filling out a questionnaire containing questions regarding the above risk factors. Furthermore, a comprehensive survey was conducted to find out whether the dog owners followed strict tick control measures earlier.

### 2.4. Laboratory Analysis

#### 2.4.1. Tick Processing

Ticks were processed in 10% KOH as per the standard procedure [7]. An Olympus microscope (100×) was used to identify the ticks at species level, considering the characteristic morphological features like hypostome, palpal segments, capitulum, scutum, and legs [6].

#### 2.4.2. Microscopic Study

Thin blood smears were prepared from capillary blood, fixed with methanol, and stained with Giemsa stain diluted 1:10 for 30 min, following the procedure of Coles [8]. Although samples were collected from 90 veterinary dispensaries, final microscopic evaluation was standardized and performed by the study team rather than independently by personnel at each dispensary. The stained blood films were examined under oil immersion at 1000× magnification for hemoparasites and ehrlichial morulae. Smears considered positive were rechecked by an experienced examiner before final classification to minimize false-positive interpretation. Parasites were identified based on characteristic morphology [7], and representative fields were photographed using an Olympus CX21 digital microscope camera.

#### 2.4.3. Multiplex PCR

All DNA extraction, PCR amplification, gel electrophoresis, and interpretation of PCR results were performed by the study team using the same standardized protocol for all samples. Genomic DNA was extracted from all collected whole-blood samples (n = 442) using the QIAamp DNA Blood Mini Kit (Qiagen GmbH, Hilden, Germany; Catalogue No. 51104; Catalogue No. 51104) according to the manufacturer’s instructions. DNA concentration and purity were assessed using a NanoDrop 1000 spectrophotometer (Thermo Fisher Scientific, Wilmington, DE, USA). DNA yield was estimated based on absorbance at 260 nm, and purity was evaluated using the A260/A280 ratio. The same DNA extracts were also used in our previous multiplex PCR-based screening study for common canine tick-borne pathogens, including *Babesia gibsoni*, *Babesia vogeli*, *Hepatozoon canis*, and *Ehrlichia canis* [9]. The present manuscript reports only the *E. canis*-focused analysis. Co-infection findings from the broader tick-borne pathogen screening were reported separately and were not included in the present analysis.

#### 2.4.4. PCR Amplification

Multiplex PCR was performed for the detection of *E. canis* using primers targeting the virB9 gene, as described by Kledmanee et al. [10], with minor modifications. Each 25-µL PCR reaction contained 12.5 µL of 2× PCR master mix containing Taq DNA polymerase, dNTPs, MgCl_2_, and reaction buffer, 1.0 µL of forward primer, 1.0 µL of reverse primer, 5.0 µL of template DNA, and 5.5 µL of nuclease-free water. The primer pair Ehr1401F and Ehr1780R was used to amplify a 380-bp fragment of the *Ehrlichia canis virB9* gene. The forward primer Ehr1401F sequence was 5′-CCATAAGCATAGCTGATAACCCTGTTACAA-3′, with a length of 30 bases, and the reverse primer Ehr1780R sequence was 5′-TGGATAATAAAACCGTACTATGTATGCTAG-3′, with a length of 30 bases. DNA from a previously confirmed *E. canis*-positive blood sample was used as the positive control, whereas nuclease-free water and DNA from a clinically healthy PCR-negative dog were used as negative controls. PCR amplification was carried out under the following cycling conditions: initial denaturation at 95 °C for 15 min; followed by 30 cycles of denaturation at 94 °C for 45 s, annealing at 65 °C for 45 s, and extension at 72 °C for 90 s; with a final extension at 72 °C for 10 min. The amplified PCR products were separated by electrophoresis on 1.5% agarose gels prepared in TBE buffer and stained with ethidium bromide. The products were visualized under UV illumination, and samples showing the expected 380-bp band were considered positive for *E. canis*.


**Primer**

**Sequence (5′-3′)**

**Length (Bases)**

**Product Size**
ForwardCCATAAGCATAGCTGATAACCCTGTTACAA30380 bpReverseTGGATAATAAAACCGTACTATGTATGCTAG30

#### 2.4.5. Sequence Analysis

Amplicons showing a clear expected 380-bp band for the *Ehrlichia canis virB9* gene were purified before sequencing using the HiPurA^®^ PCR Product Purification Kit (HiMedia Laboratories Private Limited, Thane, India), according to the manufacturer’s instructions. Purified PCR products were subjected to bidirectional Sanger sequencing using BigDye Terminator sequencing chemistry (Applied Biosystems/Thermo Fisher Scientific, Carlsbad, CA, USA) at BioServe Biotechnologies (India) Private Ltd., Hyderabad, India. The same primers used for PCR amplification, Ehr1401F and Ehr1780R, were used for the forward and reverse sequencing reactions, respectively. The electropherograms were visualized using SnapGene^®^ Viewer 4.1.9 software, and the forward and reverse sequences were assembled using the CAP3 program. The assembled sequences were edited and aligned using MEGA version 7.0. The partial nucleotide sequences of *E. canis* obtained in the present study were compared with previously reported sequences from other geographic regions available in the NCBI GenBank database. Phylogenetic analysis was performed in MEGA version 7.0 using the neighbor-joining method, and evolutionary distances were computed using the maximum composite likelihood method, expressed as the number of base substitutions per site.

#### 2.4.6. Statistical Analysis

Frequencies and percentages of risk factors and clinical findings were calculated using the PROC FREQ statement in SAS 9.4. For descriptive presentation, the clinical findings were ranked according to their observed frequency and converted to standardized scores ranging from 0 to 1 using the ceiling function Ceil (x/100, 0.1), where x represents the percentage frequency of each clinical finding. These scores were used only for descriptive ranking and were not included as variables in the logistic regression model. Age-related data was rounded off to the nearest value and classified (<1 yr. or >1 yr.) using the ROUND function. The multicollinearity test was conducted using the ‘PROC REG DATA’ statement with ‘TOL, VIF, COLLIN’ options. The tolerance was >0.5 for all risk factors and the variance inflation factor (VIF) ranged between 1.0 to 2.0, indicating no multicollinearity among the independent factors. The risk analysis was conducted using PROC LOGISTIC DATA (descending) and assigning the first value as a reference. The forest plot graph of risk factors was generated using GraphPad Prism (V 9.2.0). Significant differences were considered at *p* < 0.05. The entire statistical analysis was conducted using SAS 9.4 (Statistical Analysis Systems Institute, Inc., Cary, NC, USA).

Because microscopy and PCR were performed on the same dogs, the paired diagnostic outcomes were compared using McNemar’s test rather than Fisher’s exact test. Since culture confirmation or another independent definitive reference standard was not available, PCR was used as a molecular comparator method and not as a true gold standard. A 2 × 2 table was constructed to summarize concordant and discordant results between microscopy and PCR. Agreement between the two methods was assessed by calculating positive percent agreement, negative percent agreement, positive predictive agreement, negative predictive agreement, false-negative proportion relative to PCR, overall percent agreement, and Cohen’s kappa coefficient. These values were interpreted as relative agreement measures conditional on PCR results, rather than as absolute diagnostic sensitivity and specificity.

Positive percent agreement = TP/(TP + FN); negative percent agreement = TN/(TN + FP); negative predictive agreement = TN/(TN + FN); false-negative proportion relative to PCR = FN/(FN + TP); overall percent agreement = (TP + TN)/(TP + TN + FP + FN); positive predictive agreement = TP/(TP + FP).

Cohen’s kappa coefficient was calculated as K = (Po − Pe)/(1 − Pe), where Po = (TP + TN)/(TP + TN + FP + FN), and Pe = [((TP + FP)(TP + FN)) + ((FN + TN)(FP + TN))]/(TP + TN + FP + FN)^2^.

TP indicates samples positive by both microscopy and PCR; FN indicates samples negative by microscopy but positive by PCR; TN indicates samples negative by both microscopy and PCR; and FP indicates samples positive by microscopy but negative by PCR. Po indicates observed agreement, Pe indicates expected agreement, and K indicates Cohen’s kappa.

#### 2.4.7. Sample Size Calculation and Power of Statistical Analysis

Achieving an adequate sample size is crucial during the development of a clinical prediction model. A robust power analysis was performed utilizing the ‘statsmodels’ library within the Jupyter environment (Jupyter V. 3.0.14, Python 3.0). The inputs provided were type of data (dichotomous data), no. of predictors (eight), alpha value (0.05), and power value (80%). Notably, a small effect size (0.3) was opted to strategically evaluate its impact on determining the optimal sample size. The power analysis indicated a minimum required sample size of 348 dogs, distributed within the two groups based on Ehrlichiosis status (Yes/No). Consequently, the overall sample size of the present study (n = 442) ensures substantial statistical power, allowing for the identification of risk factors even when considering a small effect size (0.3).

## 3. Results

### 3.1. Demographics and Clinical Findings

**Figure 2:**
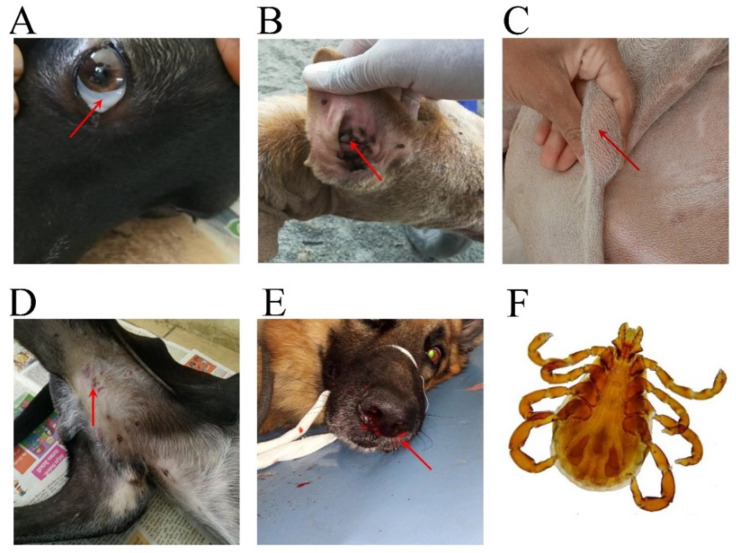
Representative clinical findings recorded in clinically suspected dogs. (**A**) Pale conjunctival mucous membrane, (**B**) tick infestation, (**C**) enlarged popliteal lymph nodes, (**D**) petechiae or ecchymoses, (**E**) epistaxis, and (**F**) *Rhipicephalus sanguineus* (brown dog tick).

The overall population characteristics of the clinically suspected dogs are presented in Table 1. Dogs were included based on clinical suspicion of canine ehrlichiosis, supported by compatible clinical findings such as inappetence, pale conjunctival mucous membranes, fever, enlarged popliteal lymph nodes, weakness, bleeding tendencies, and visible tick infestation. Tick infestation was recorded as present or absent during clinical examination and was not graded by severity. The frequency-based descriptive clinical scores are presented in Table 2, and representative clinical findings are shown in Figure 2. The most frequently recorded clinical findings included inappetence, pale conjunctival mucous membranes (Figure 2A), visible tick infestation (Figure 2B), fever, enlarged popliteal lymph nodes (Figure 2C), prolonged weakness, petechiae or ecchymoses (Figure 2D), and epistaxis (Figure 2E). Lameness, dyspnea, and limb edema were recorded less frequently. Scrotal edema, melena, hematuria, and neurological findings such as ataxia, twitching, hyperesthesia, and paresis were observed only occasionally. Visible tick infestation was recorded in 55/442 dogs (12.44%), of which 45/55 tick-infested dogs (81.82%) were PCR-positive for *E. canis*. Morphological examination of the collected ticks indicated that the predominant tick species in the study area was the brown dog tick, *Rhipicephalus sanguineus*. A representative image of the morphologically identified tick is shown in Figure 2F.

### 3.2. Diagnostic Comparison of Microscopy and PCR

**Figure 3:**
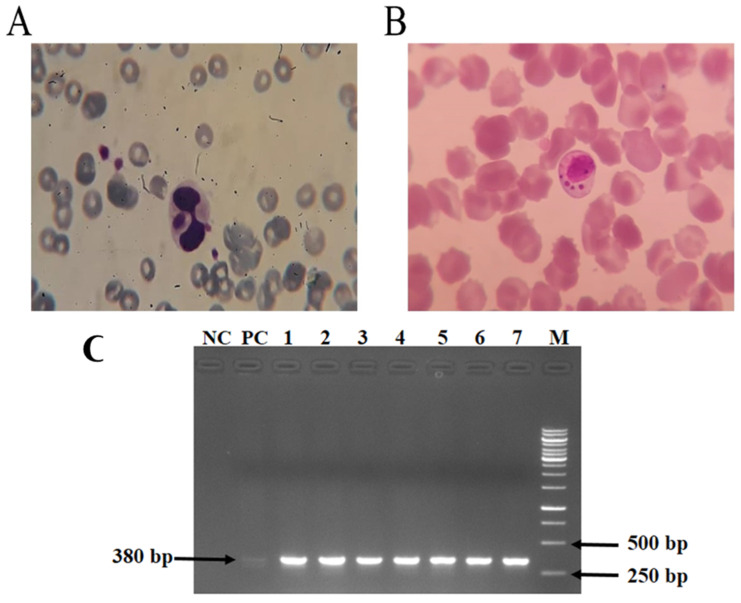
Microscopic and molecular detection of *Ehrlichia canis*. (**A**) Ehrlichial morula-like inclusion in a neutrophil, (**B**) morula-like inclusion suggestive of *Ehrlichia* infection in a mononuclear cell, and (**C**) agarose gel electrophoresis showing 380-bp amplicons of the *E. canis virB9* gene by PCR. Lane M: O’GeneRuler 1 kb DNA ladder; lanes 1–7: positive samples; NC: negative control; PC: positive control.

**Figure 4:**
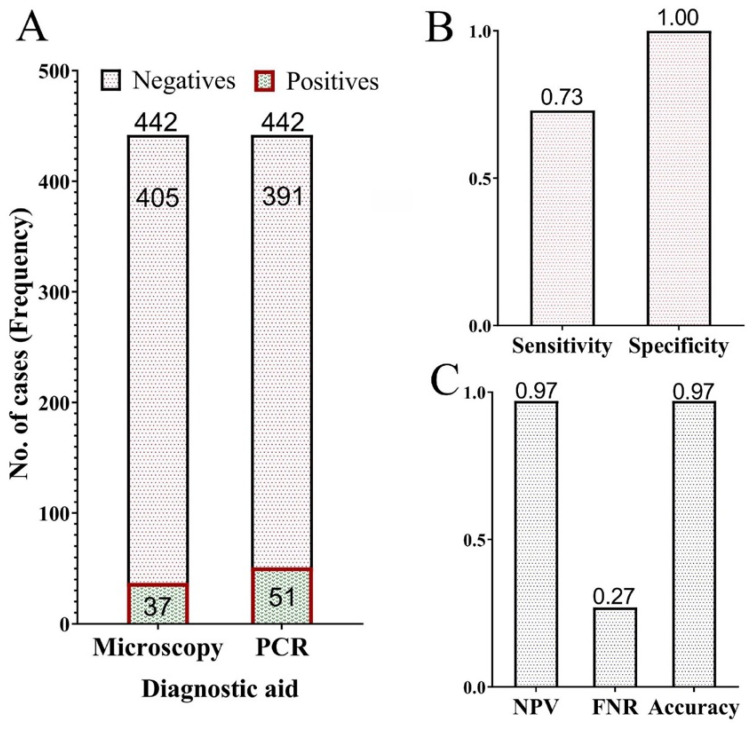
Comparison of microscopy and polymerase chain reaction (PCR) for the detection of *Ehrlichia canis* in clinically suspected dogs. (**A**) Positive and negative samples detected by microscopy and PCR. (**B**) Sensitivity and specificity of microscopy relative to PCR. (**C**) Negative predictive value (NPV), false-negative rate (FNR), and overall accuracy of microscopy relative to PCR.

Microscopic examination of peripheral blood smears revealed morulae suggestive of *Ehrlichia* infection in mononuclear cells, while PCR amplification produced the expected 380-bp band corresponding to the *E. canis virB9* gene (Figure 3A–C). A comparison of the microscopy and PCR results is shown in Figure 4. Microscopic examination identified 37/442 dogs as positive for *Ehrlichia* morulae, whereas PCR detected 51/442 dogs as positive for *E. canis*, giving an overall PCR positivity of 11.54% (Figure 4A). Because both diagnostic tests were performed on the same animals, paired diagnostic outcomes were evaluated using McNemar’s test rather than Fisher’s exact test. Using PCR as the comparator method, microscopy yielded 37 true-positive, 14 false-negative, 0 false-positive, and 391 true-negative results. Accordingly, the diagnostic performance of microscopy relative to PCR was as follows: sensitivity, 72.55%; specificity, 100%; positive predictive value, 100%; negative predictive value, 96.54%; false-negative rate, 27.45%; accuracy, 96.83%; and Cohen’s kappa, 0.824. These findings indicate that microscopy showed high specificity and strong agreement with PCR but lower sensitivity, suggesting that microscopy may miss a proportion of PCR-positive cases.

### 3.3. Risk-Factor Analysis

**Figure 5:**
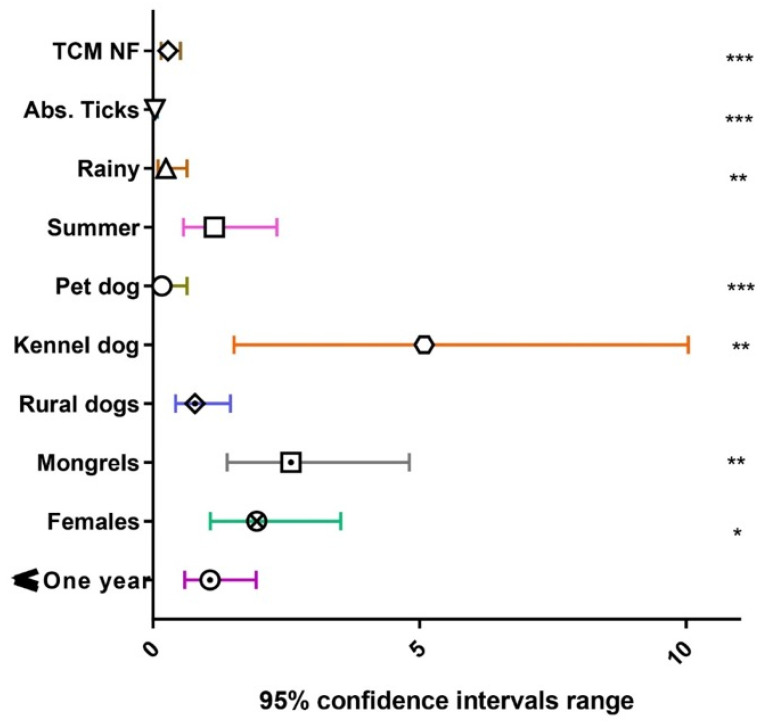
Forest plot showing odds ratios and 95% confidence intervals for factors associated with *E. canis* infection. * *p* < 0.05; ** *p* < 0.01; *** *p* < 0.001.

The risk factor analysis for *E. canis* infection in dogs is presented in Table 3, and the corresponding confidence intervals are illustrated in Figure 5. Female dogs had significantly higher odds of *E. canis* infection than male dogs (OR = 1.945; 95% CI: 1.075–3.521; *p* = 0.026), and mongrels had higher odds of infection than purebred dogs (OR = 2.587; 95% CI: 1.391–4.810; *p* = 0.002). Living condition was also significantly associated with infection. Compared with stray dogs, kennel dogs had higher odds of ehrlichiosis (OR = 5.088; 95% CI: 1.519–17.042; *p* = 0.001), whereas pet dogs had lower odds of infection (OR = 0.166; 95% CI: 0.081–0.339; *p* < 0.001). Seasonal variation was observed, with dogs sampled during the rainy season showing significantly lower odds of infection than those sampled during winter (OR = 0.244; 95% CI: 0.094–0.637; *p* = 0.001). In contrast, the odds of infection during summer were not significantly different from winter (OR = 1.152; 95% CI: 0.571–2.324; *p* = 0.693). Visible tick infestation was the strongest risk factor associated with *E. canis* infection. Using dogs with visible tick infestation as the reference category, dogs without visible tick infestation had markedly lower odds of infection (OR = 0.036; 95% CI: 0.016–0.083; *p* < 0.001). Expressed conversely, dogs with visible tick infestation had approximately 27.8-fold higher odds of infection compared with dogs without visible tick infestation. Similarly, dogs with a history of tick-control measures had significantly lower odds of infection than dogs without tick-control measures (OR = 0.281; 95% CI: 0.153–0.514; *p* < 0.001), supporting the protective association of tick-control practices.

### 3.4. Phylogenetic Analysis

**Figure 6:**
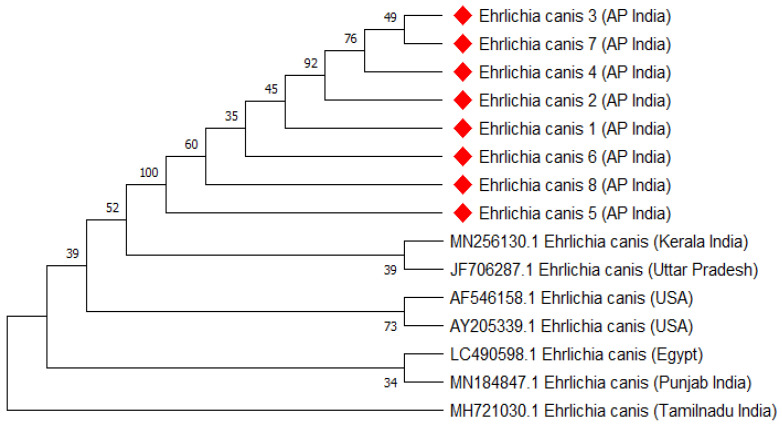
Phylogenetic tree of *Ehrlichia canis* using virB9 gene sequences.

Phylogenetic placement based on the partial *virB9* sequence showed that the representative *E. canis* sequence(s) from the present study clustered with previously reported Indian and selected global *E. canis* sequences available in GenBank, supporting molecular confirmation of the detected organism (Figure 6).

Numbers on the tree indicate bootstrap support values; only values ≥ 50% are shown.

## 4. Discussion

### 4.1. Clinical Findings

Pale conjunctival mucous membranes, fever, weakness, and visible tick infestation were common clinical findings in the present study and are consistent with the clinical presentation of canine tick-borne infections. However, these findings are not specific for *E. canis* infection and should be interpreted along with hematological and molecular diagnostic results. Enlargement of the popliteal lymph nodes may be associated with lymphoid hyperplasia resulting from persistent antigenic stimulation during ehrlichial infection. Bleeding manifestations such as petechiae, ecchymoses, and epistaxis may be related to thrombocytopenia, platelet dysfunction, or vascular injury associated with canine ehrlichiosis [11]. In the present study, bleeding tendencies were more frequently observed in German Shepherd dogs, suggesting a possible breed-related predisposition or case-composition effect that requires further investigation. Thrombocytopenia is one of the most consistent clinicopathological abnormalities in canine ehrlichiosis and may vary in severity depending on the stage and progression of infection [12]. Less frequent findings such as scrotal edema, melena, hematuria, ataxia, twitching, hyperesthesia, and paresis may occur in severe, complicated, or advanced cases, but they should not be considered specific diagnostic hallmarks of acute canine ehrlichiosis.

The distribution of clinical findings suggests that several dogs may have presented with subacute or chronic forms of canine ehrlichiosis rather than only acute infection. This is biologically plausible because dogs may show apparent recovery after the acute phase, followed by a prolonged subclinical phase that can later progress to chronic disease. Progression to chronic ehrlichiosis may be influenced by factors such as co-infections, poor immune status, persistent tick exposure, or other unmeasured clinical and pathogen-related factors [13]. Co-infection data from the same broader sample set were reported separately in our earlier publication and were therefore not included in the present manuscript. Accordingly, the present study focused specifically on *E. canis* infection, associated risk factors, microscopy–PCR comparison, and partial *virB9*-based molecular characterization.

### 4.2. Laboratory Diagnostic Methods

Giemsa-stained blood smears revealed intracytoplasmic morula-like inclusions in leukocytes. Morulae observed in mononuclear cells were considered morphologically compatible with *E. canis* infection. Inclusions observed in neutrophils were interpreted cautiously because neutrophilic morulae are more commonly associated with granulocytic ehrlichial or anaplasmal infections, although occasional reports of atypical cellular localization have been described [14].

Because of its low cost, simplicity, and accessibility, microscopy remains a useful first-line diagnostic aid in field and resource-limited veterinary settings. However, microscopy should not be considered as a definitive gold standard for *E. canis* diagnosis because its sensitivity is influenced by the stage of infection, level of circulating organisms, smear quality, and examiner expertise. In the present study, microscopy showed high specificity and positive predictive value relative to PCR, but it failed to detect 14 PCR-positive cases. The Cohen’s kappa value indicated strong, but not perfect, agreement between microscopy and PCR. These findings support the practical value of microscopy at the dispensary level while reinforcing the usefulness of PCR as a confirmatory diagnostic method, particularly in suspected cases with low parasitemia, subclinical infection, or chronic disease.

Blood smear examination may be more useful during acute infection, when circulating morulae are more likely to be detected, but it has limited sensitivity in subclinical or chronic cases [15,16]. Molecular methods, including conventional, nested, multiplex, real-time, and droplet digital PCR assays, have been developed to improve the detection and differentiation of canine tick-borne pathogens [17,18]. Several molecular targets have been used for *E. canis* detection and characterization, including 16S rRNA, *p28*, *groESL*, and other antigenic or secretion-system-associated genes [19]. In this context, the present study supports the practical use of the *virB9* gene as an additional molecular target for confirmation and regional molecular surveillance of *E. canis*. However, the diagnostic comparison should be interpreted as a field-level assessment in clinically suspected dogs rather than as evidence that PCR superiority over microscopy is a novel finding.

### 4.3. Occurrence of E. canis Infection

The PCR positivity of *E. canis* among clinically suspected dogs in the present study was 11.54%. Previous studies from different parts of the world have reported widely variable detection rates of canine ehrlichiosis, ranging from low levels to more than 50%, depending on the study population, sampling strategy, season, tick exposure, and diagnostic method used. Similar variation has also been reported within the Indian subcontinent. Higher detection rates have been reported from Tamil Nadu and Kerala, with values of 22.6% and 23.53%, respectively, reflecting regional differences in tick distribution and exposure risk [20,21]. Considerably higher positivity rates have also been reported from Indian cities such as Vijayawada, Chennai, Ludhiana, and Hissar, where reported values ranged from 30% to 50% [22,23,24].

However, detection rates across geographical regions should be interpreted cautiously because they are influenced by several confounding factors, including study design, patient selection criteria, hospital-based versus random screening, breed composition, season, landscape, sex, sampling method, and diagnostic test used. Therefore, the 11.54% PCR positivity observed in the present study should be interpreted as the occurrence of *E. canis* among clinically suspected dogs presented to veterinary dispensaries, rather than as the general population occurrence of canine ehrlichiosis in Andhra Pradesh. The main contribution of the present findings is the generation of region-specific data from a tropical area where integrated information on clinical suspicion, field-level diagnostic comparison, risk-factor assessment, and molecular confirmation remains limited.

### 4.4. Host and Environmental Risk Factors

Age was not significantly associated with *E. canis* infection in the present study. This finding is consistent with Ansari-Mood et al. [25], who reported no marked age-related difference in susceptibility among dogs. In contrast, other studies have reported age-associated variation, with higher positivity observed either in young dogs [26] or in adult dogs [27]. These differences may be related to variation in study design, sampling population, exposure history, tick-control practices, immune status, and diagnostic methods used. Similarly, the occurrence of *E. canis* infection did not differ significantly between dogs from urban and rural areas in the present study. However, previous reports have described residential-area-associated differences, with higher detection rates reported in either urban [28] or rural settings [29]. Such inconsistencies suggest that landscape alone may not be the primary determinant of infection risk. Instead, local tick burden, dog-management practices, outdoor exposure, owner awareness, and access to tick-control measures may have a greater influence on *E. canis* infection risk.

The logistic regression analysis revealed significantly higher odds of *E. canis* infection among female dogs, mongrels, and kennel dogs. The higher odds observed in females may be related to differences in physiological or reproductive status that could influence immune response and susceptibility; however, this association should be interpreted cautiously because reproductive stage and hormonal status were not specifically evaluated in the present study. The higher odds of infection in mongrels may reflect greater environmental exposure to tick vectors and less consistent use of ectoparasite-control measures rather than an inherent breed-related susceptibility. Previous studies have reported inconsistent breed-related patterns, with some showing no clear difference between breeds [30], while others reported higher susceptibility among purebred dogs [24].

Living condition also appeared to influence infection risk. The lower odds of infection observed in pet dogs may reflect better owner care, nutrition, housing, and routine ectoparasite-control practices. In contrast, kennel dogs may experience increased exposure because of close housing, shared environments, and possible maintenance of tick populations within kennel premises. Similar to the present findings, Bhattacharjee and Samrah [31] reported a lower prevalence of blood parasites in pet dogs than in stray dogs from Assam, Northeast India.

Seasonal variation was observed in the present study, although the odds of *E. canis* infection during summer were not significantly different from those during winter. In contrast, dogs sampled during the rainy season showed significantly lower odds of infection. This finding may reflect seasonal variation in the activity, feeding behavior, and development of *Rhipicephalus sanguineus*, as well as local differences in tick exposure, rainfall intensity, housing, and dog-management practices. The development and feeding activity of *R. sanguineus* are generally favored by warm conditions, whereas unfavorable environmental conditions may reduce tick activity or host contact [31]. Earlier reports have also described seasonal differences in canine ehrlichiosis, including lower detection rates during the rainy season in some regions [32].

Dogs with a reported history of tick-control measures had significantly lower odds of *E. canis* infection than dogs without such measures. This finding supports the practical importance of acaricidal treatment and routine ectoparasite control in reducing exposure to infected ticks. Overall, these associations are consistent with the established epidemiology of canine ehrlichiosis and should be interpreted as region-specific confirmation of known risk patterns rather than as evidence of new biological mechanisms. Their practical value lies in supporting targeted tick-control and surveillance strategies for dogs in Andhra Pradesh.

### 4.5. Phylogenetic Analysis

Phylogenetic analysis of the partial *virB9* sequence showed that the *E. canis* isolate from the present study clustered closely with previously reported Indian isolates, including isolates from Kerala, Uttar Pradesh, Punjab, and Tamil Nadu, as well as selected global isolates. This finding does not indicate the emergence of a distinct novel genotype; rather, it provides molecular confirmation of *E. canis* in the study region and contributes to regional sequence data. However, because the analysis was based on a short partial *virB9* fragment, the phylogenetic interpretation should be considered limited. Continued molecular surveillance using additional geographically diverse sequences, appropriate outgroup selection, multilocus sequence analysis, or whole-genome approaches would be useful to better understand the genetic diversity and geographical structuring of *E. canis* populations.

## 5. Conclusions

The present study shows that blood smear microscopy remains a valuable, accessible first-line screening tool for canine ehrlichiosis in resource-limited veterinary dispensaries. However, PCR provided a higher detection rate and should be used as a confirmatory method, particularly in clinically suspected dogs that may have low circulating organism levels. Partial *virB9*-based sequence analysis confirmed the presence of *E. canis* in the study region and supports the usefulness of this gene as a molecular target for regional surveillance. The identification of host- and management-associated factors, particularly visible tick infestation, kennel housing, and absence of tick-control measures, provides practical information for targeted surveillance, tick control, and preventive strategies in dogs from Andhra Pradesh.

## Figures and Tables

**Table 1:** Overall population characteristics of dogs.

Variable	Risk Factor	Overall Population		Positive for *E. canis*	
		Frequency	Percent	Frequency	Percent
Age	Young	172	38.91	21	12.20
	Adult	270	61.09	30	11.11
Gender	Females	208	47.06	32	15.38
	Males	234	52.94	19	8.11
Breed	Pure breed	352	79.64	32	9.09
	Mongrels	90	20.36	19	21.11
Residential setting	Urban	276	62.44	35	12.68
	Rural	166	37.56	16	9.64
Living condition	Kennel dogs	36	8.14	12	33.33
	Pet dogs	335	75.79	25	7.5
	Stray dogs	71	16.06	14	19.72
Season	Winter	86	19.46	7	8.13
	Summer	188	42.53	31	16.49
	Rainy	168	38.01	13	7.73
Ticks	Present	55	12.44	45	81.82
	Absent	387	87.56	6	1.55
Tick control measures	Not followed	356	80.54	45	12.64
	Followed	86	19.46	6	6.98

**Table 2:** Clinical score of the clinical findings observed in Ehrlichiosis.

Clinical Finding	Clinical Score
Inappetence, pale conjunctival mucous membranes	1.0
Tick infestation, fever	0.9
Enlarged popliteal lymph nodes, prolonged weakness	0.8
Petechiae or ecchymoses	0.7
Epistaxis	0.6
Lameness, dyspnea	0.5
Limb edema	0.4
Scrotal edema, ataxia, melena, twitching, hyperesthesia	0.3
Paresis, hematuria	0.2

**Table 3:** Risk factor analysis for *E. canis* infection in dogs by the logistic regression model.

Variable	Risk Factor	Chi Square Value	Odds Ratio	95% CI		Wald *p* Value
				Lower	Upper	
Age	≥One year		1			
	≤One year	0.054	1.072	0.594	1.935	0.817
Gender	Male		1			
	Females	4.960	1.945	1.075	3.521	0.026
Breed	Pure breed		1			
	Mongrels	9.510	2.587	1.391	4.810	0.002
Residential setting	Urban		1			
	Rural	0.595	0.786	0.425	1.452	0.441
Living condition	Stray dogs		1			
	Kennel dogs	7.672	5.088	1.519	17.042	0.001
	Pet dogs	29.429	0.166	0.081	0.339	<0.001
Season	Winter		1			
	Summer	0.156	1.152	0.571	2.324	0.693
	Rainy	9.401	0.244	0.094	0.637	0.001
Ticks	Present		1			
	Absent	107.917	0.036	0.016	0.083	<0.001
Tick control measures	Not followed		1			
	Followed	18.560	0.281	0.153	0.514	<0.001

## Data Availability

The original contributions presented in this study are included in the article. Further inquiries can be directed to the corresponding author.

## Abbreviations

The following abbreviations are used in this manuscript:

AP	Andhra Pradesh
CPCSEA	Committee for the Purpose of Control and Supervision of Experiments on Animals
DNA	Deoxynucleic acid
*E*. *canis*	*Ehrlichia canis*
*E. ewingii*	*Ehrlichia ewingii*
EDTA	Ethylene diamine tetra acetic acid
FN	False negative
FNR	False negative rate
FP	False positive
IAEC	Institute’s animal ethics committee
KOH	Potassium hydroxide
NCBI	National Center for Biotechnology Information
NPV	Negative predictive value
OR	Odds ratio
PCR	Polymerase chain reaction
TBE	Tris-borate-EDTA buffer
TN	True negatives
TP	True positives
UV	Ultraviolet
VIF	Variance inflation factor
